# Comparison of Maternal and Neonatal Outcomes between SARS-CoV-2 Variants: A Retrospective, Monocentric Study

**DOI:** 10.3390/jcm12196329

**Published:** 2023-10-01

**Authors:** Giosuè Giordano Incognito, Rosario Emanuele Carlo Distefano, Giorgia Campo, Ferdinando Antonio Gulino, Chiara Gulisano, Chiara Gullotta, Giuseppe Gullo, Gaspare Cucinella, Attilio Tuscano, Maria Teresa Bruno, Marco Palumbo

**Affiliations:** 1 Department of General Surgery and Medical Surgical Specialties, University of Catania, 95100 Catania, Italy; giordanoincognito@gmail.com (G.G.I.); dr.rosariodistefano@gmail.com (R.E.C.D.); giorgiacampo.96@gmail.com (G.C.); chiaragulisano@hotmail.com (C.G.); attiliotuscano@gmail.com (A.T.); mt.bruno@unict.it (M.T.B.); mpalumbo@unict.it (M.P.); 2 Unit of Gynecology and Obstetrics, Department of Human Pathology of Adults and Developmental Age, University Hospital “G. Martino”, 98100 Messina, Italy; 3 Department of Clinical and Experimental Medicine, University of Messina, 98100 Messina, Italy; chiaragullotta96@gmail.com; 4 Azienda Ospedaliera Ospedali Riuniti (AOOR) Villa Sofia Cervello, University of Palermo, 90133 Palermo, Italy; gullogiuseppe@libero.it (G.G.); gaspare.cucinella@unipa.it (G.C.)

**Keywords:** SARS-CoV-2, COVID-19, maternal outcomes, neonatal outcomes, infection

## Abstract

The impact of SARS-CoV-2 variants on maternal and neonatal outcomes during pregnancy is still poorly understood, and the emergence of different variants has further complicated our understanding of the virus’s effects. This retrospective, monocentric study aimed to fill this knowledge gap by analyzing the outcomes of pregnant women with acute SARS-CoV-2 infection caused by the Alpha, Delta, and Omicron variants. The study, conducted between December 2020 and March 2022 at San Marco Hospital, included 313 pregnant women with confirmed SARS-CoV-2 infection. The results showed that the Delta variant was associated with a significantly higher incidence of adverse outcomes, such as premature births, maternal intensive care unit admission, intrauterine growth restriction, and small for gestational age infants. Additionally, the Delta variant was linked to lower Apgar scores, higher maternal and fetal mortality rates, and increased levels of various biomarkers indicating more severe illness. Finally, the Delta variant also presented a greater possibility of vertical transmission. These findings underscore the complexity of understanding the impact of SARS-CoV-2 on pregnancy outcomes, especially considering the distinctive characteristics of different variants. By better understanding the specific impacts of each variant, appropriate preventive measures and management strategies can be implemented to optimize maternal and neonatal outcomes.

## 1. Introduction

The SARS-CoV-2 pandemic began in China’s Hubei province in December 2019, raising global health concerns due to the ease of transmission [1]. Pregnant women who contract the infection have a greater risk than non-pregnant ones of developing more severe complications, being hospitalized in the intensive care unit (ICU), and requiring mechanical ventilation [2]. They are also more likely to develop pregnancy-specific complications, such as pre-eclampsia, a premature rupture of membranes, and preterm labor [3,4]. Regarding neonatal outcomes, there was no evidence of teratogenicity of the virus when contracted in the embryonic period and no increase in the number of miscarriages was found [5]. In most cases, the virus was not transmitted vertically via the placenta; although a small percentage of placental swabs resulted positive and IgG against the viral spike protein was present in the neonatal blood. This highlights how vertical transmission is possible [6,7]. The Alpha variant was identified for the first time in September 2020. Compared to the wild-type variant, it has a higher transmissibility. It contains 17 mutations in its genome, of which 8 relate to the spike protein, leading to a greater affinity for the ACE2 receptor that the virus uses to enter cells. Furthermore, the clinical complications are also more serious than those caused by the wild-type virus [8]. The Delta variant was identified in December 2020 in India. It has a 60% higher transmission than the previous variant due to a mutation of the spike protein at the furin cleavage site. Furthermore, it has a lower sensitivity to antibodies produced by the vaccine both in vitro and in vivo [9,10,11]. Finally, the Omicron variant was identified in November 2021 in Africa and presents 30 new mutations of the spike protein with other mutations at the level of non-structural proteins. Transmissibility has undergone a further increase, but clinical features were less severe than in the previous variant [8]. Many features of pregnancy infection are still poorly understood; for example, further complications associated with SARS-CoV2 include gestational diabetes [12]. Thus, the aim of the study was to analyze the maternal and neonatal outcomes between the main three SARS-CoV-2 variants in pregnant women.

## 2. Materials and Methods

This is a retrospective, monocentric cohort study conducted at San Marco Hospital between December 2020 and March 2022. The study involved unvaccinated pregnant women with confirmed acute SARS-CoV-2 infection, as determined by positive time quantitative reverse transcription PCR (qRT-PCR) nasopharyngeal swabs. Female patients with physiologic pregnancies and spontaneous conception, with an age between 18 and 44 years old, were included in the study. Moreover, patients with previous/current history of obstetrics pathologies (such as preeclampsia and/or gestational diabetes) under control before the infection, as well as those with physiologic twin pregnancies, were also included in the analysis. Patients with disease that was not under control were not included, in order to eliminate any confounding bias. Accordingly, women vaccinated against SARS-CoV-2 and those with previous or obstetric pathologies that could have altered the significance of the results, monochorionic monoamniotic twin pregnancies, multiple pregnancies, assisted reproductive technology (ART) conception, and women under 18 years were excluded due to their high risk of complications.

Patients with negative qRT-PCR nasopharyngeal swabs at the time of delivery were excluded if they did not show any signs or symptoms of the disease, because they were considered and managed as physiologic pregnancies. In total, 313 pregnant women with proven acute SARS-CoV-2 infection were enrolled. Among them, 104 patients with positive qRT-PCR nasopharyngeal swabs from December 2020 and May 2021 were included in the Alpha group, 55 positive ones between July 2021 and December 2021 were included in the Delta group, and finally, 154 positive patients between January 2022 and March 2022 were enrolled in the Omicron group.

Personal, obstetric, and biochemical data were obtained from the medical records and the “Modulab” laboratory management system. The possibility of vertical transmission was assessed through SARS-CoV-2 RT-PCR placental swabs. For each of the three variant groups the following items were analyzed: the average age of the patients, the average number of pregnancies and previous types of births, the average gestational age at the time of delivery, the percentage of admissions in the II and III trimesters, deliveries in the II and III trimesters, percentage of the type of delivery, i.e., vaginal delivery (VD) or cesarean section (CS), percentage of admission to intensive care unit (ICU), maternal and neonatal death, mean of neonatal weight, mean of Apgar at 1 and 5 min, mean of the time interval between the diagnosis of SARS-CoV-2 infection and childbirth, an average of biochemical parameters recorded for the entire period of patients’ hospitalization, i.e., C-reactive protein (CRP), procalcitonin (PCT), interleukin 6 (IL-6), hemoglobin (Hb), leukocytes with leukocyte formula including neutrophils and lymphocytes, D-dimer, aspartate aminotransferase (AST), and alanine aminotransferase (ALT), percentage of positive and negative placental swabs.

Continuous variables were presented as means ± standard deviations (SD). Categorical variables were summarized as percentages. A *p*-value of less than 0.05 was established as the threshold for determining statistical significance.

The study was conducted in accordance with the Declaration of Helsinki and approved by the Ethics Committee of the University of Catania (Code number 01/163 and approval date of 10 January 2022). Each woman received appropriate counseling about the purpose of the research and the guarantee of anonymous treatment of personal data, according to Italian laws guaranteeing privacy, and signed an informed consent form for data collection. 

## 3. Results

### 3.1. Patient Baseline Characteristics

The average age of the patients was similar in the three groups (Alpha = 31.5 ± 4.7 years vs. Delta = 31.4 ± 5.1 years vs. Omicron = 31.7 ± 4.5 years, *p* = 0.942), as well as the average number of previous pregnancies (1.7 (1.0–2.6) vs. 1.6 (0.9–2.4) vs. 1.5 (0.8–2.3), *p* = 0.729), the average number of previous miscarriages (0.3 ± 0.5 vs. 0.2 ± 0.4 vs. 0.3 ± 0.5, *p* = 0.664), the type of previous deliveries (74.4% VD and 25.6% CS vs. 73.6% VD and 26.4% CS vs. 72.2% VD and 25.8% CS, *p* = 0.978) and the mean time interval between the diagnosis and the time of delivery (8.5 ± 3.2 days vs. 8.3 ± 3.5 days vs. 8 ± 3.1 days, *p* = 0.863). Delta variant was associated with a lower gestational week at the time of delivery (38 ± 1.4 vs. 36 ± 1.8 vs. 37.7 ± 1.6, *p* < 0.001) and a higher percentage of deliveries in the II trimester (2% vs. 5.8% vs. 2.4%, *p* = 0.049). The Alpha group gave birth more frequently by means of CS (61.6%), while the lowest rate was recorded among women in the Omicron group (32.5%). 46.3% of patients in the Delta group gave birth by means of CS. Patients’ baseline characteristics are illustrated in Table 1.

### 3.2. Biochemical Data

The Delta variant was correlated with higher CRP (56.93 ± 20.12 vs. 107.13 ± 40.34 vs. 59.51 ± 21.46, *p* < 0.001), PCT (0.20 ± 0.08 vs. 6.47 ± 2.51 vs. 0.22 ± 0.09, *p* < 0.001), IL-6 (30.21 ± 10.13 vs. 339.54 ± 100.51 vs. 24.13 ± 9.31, *p* < 0.001), D-dimer (1257.31 ± 401.13 vs. 1594.58 ± 510.67 vs. 1279.79 ± 412.37, *p* < 0.001), AST (54 ± 15.32 vs. 100 ± 30.13 vs. 59 ± 16.42, *p* < 0.001) and ALT (61 ± 17.32 vs. 116 ± 33.46 vs. 65 ± 18.23, *p* < 0.001) levels and lymphocyte rates (15.32% ± 4.51 vs. 17.57% ± 5.12 vs. 15.86% ± 4.73, *p* = 0.032) and lower Hb levels (11.15 ± 1.51 vs. 9.79 ± 1.85 vs. 11.59 ± 1.45, *p* < 0.001). Leukocyte levels (10.38 ± 2.13 vs. 10.89 ± 2.34 vs. 10.95 ± 2.28, *p* = 0.572) and neutrophil rates (74.15% ± 6.31 vs. 79.66% ± 7.24 vs. 78.55% ± 6.91, *p* = 0.061) were similar among the three groups. Biochemical data are displayed in Table 2.

### 3.3. Maternal and Neonatal Outcomes

Nine Delta women were admitted to the ICU for worsening respiratory symptoms, while none of the Alpha group and only two Omicron patients were transferred. None of the women in the Alpha and Omicron groups died, while one Delta woman died. The Delta variant was most frequently associated with small for gestational age (SGA) newborns (with an average infant weight of 2533 ± 513.62 g vs. 3252 ± 412.32 g of the Alpha group and 3161 ± 402.18 g of the Omicron patients, *p* < 0.001) and with the lowest 1st minute and 5th minute Apgar scores (*p* < 0.001). The only three neonatal deaths were observed in the Delta group. Finally, this variant was associated with higher placental swab rates (5% vs. 20% vs. 7%, *p* < 0.001). Maternal and neonatal outcomes are reported in Table 2.

## 4. Discussion

The research and discussions prompted globally by the COVID-19 pandemic have been more extensive than any previous pandemic [13], highlighting an unprecedented global resonance and deep-rooted anxieties, especially in sensitive fields such as obstetrics.

The present study demonstrates the greater severity of the infection by the SARS-CoV-2 Delta variant compared with Alpha and Omicron variants in both pregnant women and newborns.

The Delta variant became the dominant strain in many regions around the world within a few months of its discovery, signaling its heightened transmissibility compared to earlier variants. It has been increasingly clear that the Delta variant was not only more transmissible but potentially more severe, causing higher rates of hospitalization and complications in infected individuals, including those who were pregnant with an increase in adverse maternal and fetal outcomes in pregnancies affected.

The Alpha variant was associated with the highest rate of CS. Nevertheless, this was probably related to the fact that at the beginning of the pandemic period, there was little knowledge on how to manage the infected patients and CS was preferred due to worse obstetric outcomes compared to uninfected women. Subsequently, the number of CS decreased among the Delta patients, despite worse both maternal and neonatal morbidities. With the Omicron variant, a further decrease of CS occurred thanks to a better knowledge of infection.

In order to better understand the data regarding the effect of the infection on the lab values analyzed, the authors also reviewed the current literature on their physiologic changes during pregnancy. During pregnancy, CRP values may be physiologically higher and may be related to the inflammatory processes that support the pregnancy itself, implantation, and the onset of labor. Significantly elevated or permanently elevated values should be carefully evaluated. In fact, in the middle months of gestation, quiescence of the inflammatory state and immune activation is expected. At this stage, high CRP values seem to be associated with an unfavorable evolution of gestation for both mother and fetus, due to the increased risk of pre-eclampsia, preterm delivery, and low birth weight of the newborn [14]. PCT is a biological marker of sepsis, septic shock, and severe inflammatory reactions. The finding of elevated serum PCT values is indicative of an inflammatory response to a systemic bacterial infection. It does not undergo physiological changes during pregnancy [14]. IL-6 is a protein produced by the immune system, involved in the regulation of the immune response, and its values during pregnancy are considered comparable in this case too [14]. During gestation, physiological dilution anemia can be found, linked to the fact that the total volume of blood increases by about 1.25 L, due to both the increase in plasma content and the increase in erythropoiesis in the medulla. However, the erythrocyte mass does not increase in parallel, and therefore an apparent reduction in Hb levels results. Even following both vaginal and cesarean births, hemoglobin may be reduced because of the blood losses that occur. The women will therefore have hemoglobin values of around 11 g/dl and hematocrit of around 34% [14]. Throughout pregnancy, leukocytes may reach 11,000/mm^3^ in the first weeks and increase up to 20,000/mm^3^ in the last trimester. However, the values increase further during labor and can even reach 30,000 leukocytes/mm^3^. In particular, the neutrophils undergo the greatest increase because they are the first congenital line of defense that protects both the fetus and the mother. Conversely, lymphocytes undergo a physiological reduction, mainly due to hormonal changes and the increase in total body fluids that occurs during gestation, which leads to hemodilution with a slight decrease in all blood cells, especially lymphocytes [14]. With reference to D-dimer, it increases its levels during the first weeks of gestation, continuing even in the postpartum period, regardless of the type of delivery [15,16]. It can reach values above 4000 ng/mL during pregnancy in the third trimester [17]. As for the transaminases, the normal values of AST vary between 8 and 48 U/L, while for ALT they range between 7 and 55 U/L. During pregnancy, an increase in their value could be physiological because of an increase in liver activity, and hormonal changes could alter their value [14].

The Delta group was associated with worse biochemical changes than the other two variants. The almost double CRP and much higher IL-6 values indicate that the pro-inflammatory picture is much more pronounced during the infection with this variant. PCT values were much higher in this group, underlining how the probability of bacterial superinfection, which further complicates the clinical picture of patients, is greater for the Delta variant infection than for Alpha and Omicron. Higher levels of D-dimer could be correlated with an increased risk of developing thrombotic phenomena. Finally, high AST and ALT values indicate a greater likelihood that patients infected by the Delta variant have liver damage. Clinically, the laboratory values do not influence management of the pregnant patients, but these values could be an expression of a higher general serum inflammation, or, from another point of view, of reduced attention of pregnant people to SARS-CoV-2 infection, which could have determined a more severe Delta variant.

Delta women were associated with worse maternal morbidity and the only maternal death occurred in this group due to postpartum respiratory failure. A higher rate of neonatal morbidity was found in the Delta variant and the three neonatal deaths were all born prematurely by emergency CS for acute fetal distress: two were born at 28 gestational weeks weighing 1195 g and 1050 g, respectively, and the third was born at 35 gestational weeks weighing 1820 g. Finally, the study of placental swabs on both maternal and fetal sides indicates that the probability of vertical transmission is considerably higher for the Delta variant than for the other two. Some previous studies confirmed the results of our work. A retrospective cohort study conducted at the University of Alabama in Birmingham [18] states that there was a higher incidence of more severe clinical conditions in patients with the Delta variant, rather than Pre-delta and Omicron, as well as a higher admission rate to the ICU. In addition, they were more frequently subjected to pharmacological treatments, ventilatory support, or intubation and had a higher incidence of thromboembolic phenomena. There was also a higher frequency of preterm deliveries, emergency CS due to the worsening of maternal clinical conditions, and neonatal admissions to the neonatal intensive care unit. Another cohort study, conducted on 61 patients at the University of Texas Medical Branch [19], found that patients with Delta infection had a lower mean gestational age at the time of diagnosis or onset of symptoms than the previous variants and were more likely to be symptomatic. Higher morbidity in this group was also described at Parkland Hospital, particularly where vaccine acceptance is low [20]. The mortality rate of pregnant women in Brazil was double (15.6%) than it was during the former Alpha variant (7.4%) [21]. Furthermore, the intrauterine fetal mortality rate was higher in Delta patients [22]. Finally, Guan et al. confirmed that the main laboratory alterations in COVID-19 pregnant women concern proinflammatory cytokines and appear to be greatest in patients with the Delta variant [23]. Considering the Delta variant, maternal death and preterm birth <37 weeks were, respectively, 0.63% (95% CI, 0.05−1.20%), and 18.58% (95% CI, 9.52−27.65%) [24]. In a previous study of our research group [25] and in some other scientific works [26,27,28,29,30,31,32] Reactive C protein (CRP) serum levels were higher than the normal range, corresponding to a mean value of 56.93 ± 49.57 mg/L. COVID-19 infection in pregnant women seems to negatively affect both maternal and neonatal outcomes.

Our study presents several strengths in its investigation of the impacts of different SARS-CoV-2 variants on maternal and neonatal outcomes during pregnancy. One notable strength is the inclusion of a relatively large sample size, with 313 pregnant women confirmed to have SARS-CoV-2 infection. This sizable cohort provided a substantial dataset for analysis, enhancing the statistical power of the study. Another strength lies in the study’s focus on physiologic pregnancies, which ensures a specific target population for analysis. By excluding individuals with uncontrolled obstetric pathologies, the authors aimed to reduce confounding biases and isolated the effects of the viral variants on pregnancy outcomes. Additionally, the study examined a range of crucial outcomes, including premature births, ICU admission, intrauterine growth restriction, and biomarker levels. By considering multiple endpoints, the study offers a comprehensive assessment of the impacts of different variants on both maternal and neonatal health. However, it is important to acknowledge the limitations of the manuscript. One significant limitation is the absence of variant typing based on molecular analysis. Instead, the study relied on the timing of maternal infection to categorize the SARS-CoV-2 variants. This approach introduces uncertainty, as the groups identified based on timing may not represent pure variant groups. It is possible that the reported findings may have been influenced by the presence of mixed variants within the identified groups, potentially impacting the accuracy of the results. Furthermore, the retrospective design of the study presents inherent limitations. The reliance on existing medical records may have introduced biases and incomplete data, leading to potential confounding factors that could impact the validity of the findings. Additionally, the single-center nature of the study may limit the generalizability of the results to a broader population. Variations in patient demographics, healthcare resources, and management protocols across different centers may affect the external validity of the findings. The study’s exclusion criteria also pose limitations. While focusing on physiologic pregnancies is a strength, excluding vaccinated individuals and those with specific obstetric conditions might have introduced selection bias and limited the generalizability of the findings. The decision to exclude vaccinated people was finalized to obtain a more homogeneous sample of patients; furthermore, considering the period analyzed for infection (December 2020 to March 2022) most of the pregnant people were not vaccinated because there were no vaccinations or because most of them declined the invitation to have a vaccination. It is important to consider these limitations when interpreting the results, as they may restrict the applicability of the findings to broader populations. The limitations of our study include its retrospective design and the fact that the study did not consider symptom recurrence. In addition, the analysis of fertility and live birth outcomes was limited to our medical-assisted conception and maternity units’ databases, whereas pregnancy and live birth data for patients who might have moved out of our hospital’s catchment area could be missing. 

## 5. Conclusions

The Delta variant is associated with more unfavorable maternal and neonatal outcomes than the Alpha and Omicron variants. In fact, this variant is associated with a higher incidence of preterm births, SGA infants, lower Apgar scores, higher maternal and fetal mortality, higher maternal admission to ICU, higher CRP levels, as well as PCT and IL-6, higher levels of lymphocytes, D-dimer, and transaminases. It is also associated with a higher rate of placental SARS-CoV-2 detection. Further research with a larger and more diverse population, encompassing multiple centers and incorporating variant typing, would help validate the results and provide a more comprehensive understanding of the effects of SARS-CoV-2 variants on maternal and neonatal health.

## Figures and Tables

**Table 1:** Patients’ baseline characteristics.

	Alpha Group (n = 104)	Delta Group (n = 55)	Omicron Group (n = 154)	*p*-Value
Mean age (years)	31.5 ± 4.7	31.4 ± 5.1	31.7 ± 4.5	0.942
Mean previous pregnancies (n)	1.7 (1.0–2.6)	1.6 (0.9–2.4)	1.5 (0.8–2.3)	0.729
Mean previous miscarriages (n)	0.3 ± 0.5	0.2 ± 0.4	0.3 ± 0.5	0.664
Type of previous deliveries (%)	VD 74.4, CS 25.6	VD 73.6, CS 26.4	VD 72.2, CS 25.8	0.978
Mean gestation period (weeks)	38 ± 1.4	36 ± 1.8	37 ± 1.6	<0.001
Interval Diagnosis–Delivery (days)	8.5 ± 3.2	8.3 ± 3.5	8 ± 3.1	0.863
II trimester at delivery (%)	2.0	5.8	2.4	0.049
III trimester at delivery (%)	98.0	94.2	97.6	0.049
Type of delivery (%)	VD 38.4, CS 61.6	VD 53.7, CS 46.3	VD 67.5, CS 32.5	0.003

Abbreviations: %: percentage; CS: cesarean section; Interval Diagnosis–Delivery: mean interval between the diagnosis of maternal COVID-19 infection and the date of delivery; n: number; VD: vaginal delivery. Continuous variables are expressed as mean ± standard deviations (SD) and categorical variables were summarized as percentages.

**Table 2:** Biochemical data and maternal-neonatal outcomes.

	Alpha Group (n = 104)	Delta Group (n = 55)	Omicron Group (n = 154)	*p*-Value
CRP (mg/L)	56.93 ± 20.12	107.13 ± 40.34	59.51 ± 21.46	<0.001
PCT (ng/mL)	0.20 ± 0.08	6.47 ± 2.51	0.22 ± 0.09	<0.001
IL-6 (pg/mL)	30.21 ± 10.13	339.54 ± 100.51	24.13 ± 9.31	<0.001
Hb (g/dL)	11.15 ± 1.51	9.79 ± 1.85	11.59 ± 1.45	<0.001
Leukocytes (n/mm^3^)	10.38 ± 2.13	10.89 ± 2.34	10.95 ± 2.28	0.572
Neutrophil (%)	74.15 ± 6.31	79.66 ± 7.24	78.55 ± 6.91	0.061
Lymphocyte (%)	15.32 ± 4.51	17.57 ± 5.12	15.86 ± 4.73	0.032
D-dimer (ng/mL)	1257.31 ± 401.13	1594.58 ± 510.67	1279.79 ± 412.37	0.001
AST (U/L)	54 ± 15.32	100 ± 30.13	59 ± 16.42	<0.001
ALT (U/L)	61 ± 17.32	116 ± 33.46	65 ± 18.23	<0.001
Maternal admission to ICU (n)	0	9	2	0.002
Maternal mortality (%)	0.0	1.8	0.0	0.049
Neonatal weight (grams)	3252 ± 412.32	2533 ± 513.62	3161 ± 402.18	<0.001
Apgar score at 1st min	8.84 ± 1.13	8.18 ± 1.41	8.75 ± 1.19	<0.001
Apgar score at 5th min	9.84 ± 0.67	9.26 ± 0.88	9.80 ± 0.71	<0.001
Neonatal mortality (%)	0.0	5.5	0.0	0.001
Vertical transmission (%)	5.0	20.0	7.0	<0.001

Abbreviations: ALT: alanine aminotransferase; AST: aspartate aminotransferase; CRP: C-reactive protein; Hb: hemoglobin; ICU: intensive care unit; IL-6: interleukin 6; n: number; %; PCT: procalcitonin. Continuous variables are expressed as mean ± standard deviations (SD) and categorical variables were summarized as percentages.

## Data Availability

Not applicable.
